# The impact of a supranetwork multidisciplinary team (SMDT) on decision-making in testicular cancers: a 10-year overview of the Anglian Germ Cell Cancer Collaborative Group (AGCCCG)

**DOI:** 10.1038/s41416-020-01075-1

**Published:** 2020-09-29

**Authors:** Jonathan Shamash, Wendy Ansell, Constantine Alifrangis, Benjamin Thomas, Peter Wilson, Sara Stoneham, Danish Mazhar, Anne Warren, Tristan Barrett, Susanna Alexander, Sarah Rudman, Michelle Lockley, Daniel Berney, Anju Sahdev

**Affiliations:** 1 Barts Health NHS Trust, London, UK; 2 University College London Hospitals, London, UK; 3 Cambridge University Hospitals NHS Foundation Trust, Cambridge, UK; 4 Norfolk and Norwich University Hospitals NHS Foundation Trust, Norwich, UK; 5 Guy’s & St Thomas’ NHS Foundation Trust, London, UK; 6 Centre for Cancer Cell and Molecular Biology, Barts Cancer Institute, Queen Mary University of London, London, UK

**Keywords:** Germ cell tumours, Health policy

## Abstract

**Background:**

The germ cell supranetwork multidisciplinary team (SMDT) for the Anglian Network covers a population of 7.5 million.

**Methods:**

We reviewed 10 years of SMDT discussion and categorised them into five domains ((1) overall outcome, (2) chemotherapy regimens—untreated disease and salvage therapy, (3) radiology, (4) pathology and (5) complex cases) to assess the impact of the SMDT.

**Results:**

A total of 2892 new cases were reviewed. In the first 5 years, patients with good prognosis disease had poorer survival in low-volume vs high-volume centres (87.8 vs 95.3, *p* = 0.02), but the difference was no longer significant in the last 5 years (93.3 vs 95.1, *p* = 0.30). Radiology review of 3206 scans led to rejection of the diagnosis of progression in 26 cases and a further 10 cases were down-staged. There were 790 pathology reviews by two specialised uropathologists, which lead to changes in 75 cases. ^18^F-fluorodeoxyglucose (^18^FDG) PET-CT was undertaken during this time period but did not help to predict who would have viable cancer. A total of 26 patients with significant mental health issues who were unable to give informed consent were discussed.

**Conclusion:**

SMDT working has led to an improvement in outcomes and refining of treatment in patients with germ cell tumours.

## Background

Disease-specific multidisciplinary team (MDT) working has been championed as a way of improving outcomes for complex conditions. It has long been recognised that patients treated in cancer centres encountering more cases of a particular condition tend to have better outcomes.^1,2^ Testicular tumours are relatively rare—many cases present with stage I disease and can be managed using a strategy of surveillance with or without adjuvant chemotherapy.^3^ Some patients present with low-volume metastatic disease (International Germ Cell Cancer Collaborative group (IGCCCG) good prognosis)^4^ and can be treated with short-course combination chemotherapy. Much less commonly patients present with advanced disease—either with very high tumour markers and/or adverse metastatic sites (IGCCCG poor prognosis).^4^

The Anglian Germ Cell Cancer Collaborative Group (AGCCCG) was formed in response to the “Improving Outcomes Guidance”^5^ and links nine hospitals covering a population of 7.5 million people. The group strives to improve and standardise outcome regardless of the hospital to which the patient presents. Treatment for good and some intermediate prognosis patients is administered in low-volume cancer centres within the AGCCCG. Low-volume centres (LVCs) were defined as those where <30 new cases were seen per year. If a greater number were seen, the centres were defined as high volume. Poor prognosis patients, those who relapse following multi-agent chemotherapy and those for whom standard cisplatin-based therapy is contra-indicated have care centralised at one of the three larger hospitals. Surgery for residual disease is centralised: retroperitoneal dissection carried out at one of the two centres and thoracic, hepatic or neurosurgery when required is undertaken at the appropriate specialist centre.

The Group aims to harmonise treatments and follow-up strategies and to ensure patient access to clinical trials.

Although this is widely regarded as a “testicular” supranetwork multidisciplinary meeting (SMDT), it is a “germ cell” SMDT, and as such, all germ cell patients are discussed, including those with mediastinal and extra-gonadal primaries and females with germ cell tumours. Female germ cell tumours and teenage and young adults (TYA) with germ cell tumours have been discussed since 2012.

## Methods

The Group was founded on the principles annotated within the IOG (improving outcomes guidance).^5^ There are core members and extended members—the extended members being oncologists from smaller cancer units and surgeons who perform infrequent operations for this group of patients (thoracic, hepato-biliary and neurosurgeons). The inclusion of TYA specialists has allowed for compliance with IOG CYP (improving outcomes guidance for children and young people with cancer).^6^

There is a standardised operating procedure for the SMDT detailing treatment protocols, follow-up schedules, appropriate chemotherapy regimens and age-appropriate trials available within the network. Patients needing emergency treatment are discussed outside the meeting with the Chair so that initial therapy is not delayed. Originally the Group met every 2 weeks but in the last 6 years meetings have become weekly. The meetings are teleconferenced to include all specialist centres. All radiology and pathology are centrally reviewed unless the radiology or pathology for stage I disease were initially reported by a specialist radiologist or pathologist, respectively, from a high-volume centre (HVC).

Documentation of all SMDT decisions is collated and verified by the Chair at the host hospital and is sent back to all participating hospitals within 3 days.

All new patients are discussed at the meeting. However, to optimise time for discussion of complex patients, the SMDT agreed that the stage I patients who follow protocol are only discussed briefly, registered and all their risk stratifying data are collected (vascular invasion, tumour size, rete testis invasion).

Patients discussed in detail (including formal radiological review) are all relapsed patients, patients on completion of chemotherapy and post retroperitoneal lymph node dissection (RPLND) and any in follow-up with whom there is a concern. This was not specified in IOG, but the Group deemed this of particular importance in germ cell patients, where cure is the goal even following multiple lines of treatment, and it adds to the global learning for rarer situations and disease presentations. In 2019, this recommendation was added to the updated guidance by NHS England for the management of specialised testicular cancer services.^7^

To ensure good governance, an annual meeting is held during which the operational policy is reviewed, recommendations for minimum follow-up and scanning schedules are agreed, clinical trials updates are presented and new protocols in development are discussed. Annual audits of outcome are also reviewed, with morbidity and mortality, together with audits of supportive therapies.

### Data collection

A specific proforma was developed for patient inclusion for discussion, to be submitted prior to the SMDT. These forms provided the basis for this publication. As a large amount of data was collected, this was grouped into different domains in order to allow meaningful interpretation. The meetings between January 2007 and 2017 were reviewed in detail as this represented the time where the more detailed proforma was used. The outcome data for the HVCs vs LVCs is taken from January 2006 and 2016 to ensure that there was at least 2 years of outcome available as basic data regarding survival and progression was clearly available using the old proforma.

Statistical analyses were performed with the STATA/SE version 16.1 statistical software package. Overall survival (OS) and progression-free survival (PFS) were assessed using the Kaplan–Meier method, and log-rank test was used to compare the progression-free interval and the OS between the categorical groups. Patients were censored at the time of last follow-up.

### Clinical trials

Participation in clinical trials was actively encouraged for patients. Some national studies were open at most centres, but the phase 2 studies required patients to travel to one of the HVCs.

## Results

A summary of the overall results of supranetwork work is split into five domains.

1. Overall outcomes
2. Chemotherapy regimens—untreated disease and salvage therapy
3. Radiology
4. Pathology
5. Specialised cases

### Domain 1: overall outcomes

In the 10-year period, the SMDT discussed 2892 new cases of germ cell tumours with a total of 5365 discussions. The overall number of significant decisions made to patient management by the SMDT was 6.4%. Chemotherapy regimen was altered for 36 patients: 22 patients received less and for 14 patients it was increased. Surgery was added for 101 patients while a change to re-scan +/− watch and wait was instigated for 111 patients.

SMDT management decisions that did not fit standard criteria were required for 41 patients; 25 were complex discussions due to multiple recurrences or other co-morbidities; 13 vulnerable adults had tailored treatment recommendations. A further 6 patients were found to be suitable for trial participation as a result of SMDT discussion. In addition, pathology changes were made for 9.4% and radiology 3.4%.

### Outcomes of patients treated for metastatic disease reported by centre size and changes over time

The outcomes were analysed by centre size and over time. The first 5 years and then the last 5 years have been analysed separately to see if there were any differences attributable to the effect of SMDT working—see Table 1. The median follow-up for this period was 51.1 months, while the median follow-up for the last 5 years was 36.4 months.

**Table 1:** Outcomes of patients with metastatic disease treated in AGCCCG SMDT: analysed by time^a^.

		2006–2010	2011–2015			2006–2010	2011–2015	
	*N*	PFS (2 years)	PFS (2 years)	*p* value	*N*	OS (5 years)	OS (5 years)	*p* value
Good	265	89.8%	93.0%	0.17	290	93.7%	94.7%	0.196
Intermediate	50	68.6%	71.8%	0.52	38	67.9%	83.6%	0.363
Poor	57	65.2%	50.6%	0.095	59	69.0%	59.2%	0.67

^a^This includes patients with missing/unclear histology but allocated to an IGCCCG prognostic group (*N* = 4).

Overall, for good and intermediate prognosis patients, there has been an improvement in outcome as assessed by PFS and OS. While the numbers for the PFS and OS of poor prognosis patients appear to have deteriorated, this was not statistically significant (*p* values 0.095 and 0.675, respectively).

As patients with good-risk disease could be treated either at HVCs or LVCs, the results for each 5-year period were compared. See Table 2.

**Table 2:** Outcomes of patients with good prognosis metastatic disease treated in AGCCCG SMDT: analysed by time and centre.

	2006–2010			2011–2015		
	HVC	LVC	*p* value	HVC	LVC	*p* value
	*N* = 197	*N* = 68		*N* = 249	*N* = 41	
PFS (2 years)	90.7%	86.8%	0.21	93.9%	76.9%	0.24
OS (5 years)	95.3%	87.8%	0.021	95.1%	93.3%	0.304

In the first 5 years, there was a statistically significant difference in the OS with men doing less well if treatment was given in the LVCs. In the second 5 years, there was no statistical significance in the outcomes.

There was a trend to increase in OS overall, with the HVCs remaining stable (from 95.3% to 95.1%) and the LVCs increasing from 87.8% to 93.3%.

### Domain 2: chemotherapy regimens

The SMDT agreed a set of regimens for use—standard BEP (bleomycin, etoposide and cisplatin) 3 cycles or EP (etoposide and cisplatin) 4 cycles were available at all treating centres for good prognosis disease.^8–10^ Indications to omit bleomycin varied between centres but agreement was reached to limit the deviation from BEP chemotherapy where possible. Two centres offered carboplatin monotherapy (carboplatin AUC 10)^11^ for metastatic seminoma, and it was agreed that patients from smaller centres could choose to be referred for this option if they wished—the outcomes for seminoma good prognosis treated with carboplatin or combination chemotherapy are shown in Table 3. Carboplatin was initially used as part of a clinical study—it was then extended to patients with metastatic seminoma who had a contra-indication to standard BEP—increasingly, it has been used as an alternative to BEP as the data have continued to mature. All such patients were aware that in prior randomised studies single-agent carboplatin had been inferior to combination treatment but that the dose that was being offered had shown comparable though non-randomised outcomes.

**Table 3:** Seminomas good prognosis treated in AGCCCG SMDT^a^.

Monotherapy (*N* = 108)		Combination therapy (*N* = 147)		
Median age	39	Median age	40.2	*p* value
PFS (2 years)	90.4%	PFS (2 years)	89.8%	
OS (5 years)	92.7%	OS (5 years)	96.8%	0.726

^a^Four patients are not recorded as either monotherapy or combination therapy.

Treatment for those with poor prognosis disease was centralised. Adult patients treated at Barts and TYA patients treated at UCLH were offered an intensive regimen—GAMEC (granulocyte colony-stimulating factor, actinomycin, methotrexate, etoposide and cisplatin).^12^ This was given either initially or following induction therapy. This regimen was chosen as it had been developed within the network as part of a clinical trial and it had shown higher PFS and OS than had been shown with BEP in similar patients. The other centres used BEP or VIP (etoposide, ifosfamide and cisplatin) if bleomycin was contra-indicated.^10,13^ The results in terms of PFS and OS are shown. There is a clear improvement in PFS associated with the use of an intensive regimen in fit patients. OS is similar as salvage therapy following BEP or VIP is more likely to be effective. See Table 4.

**Table 4:** IGCCCG poor prognosis treated in AGCCCG SMDT.

Centre employing intensive therapy (GAMEC)	Centres using standard 3 weekly therapies		
	*N* = 69	*N* = 47	*p* (log rank)
PFS (2 years)	64.3%	55.0%	0.266
OS (5 years)	64.6%	69.3%	0.582

### Salvage therapy

Salvage therapy was provided in one of the two centres, with high-dose chemotherapy confined to one centre for adults and one for TYA. Overall salvage therapy used conventionally dosed therapy with high-dose chemotherapy reserved for the third-line setting, unless patients had relapsed following an intensive regimen (GAMEC,^12^ POMB-ACE^14^ or CBOP-BEP^15^) when high-dose chemotherapy was performed as part of initial salvage therapy (second line). See Table 5.

**Table 5:** Subsequent high-dose chemotherapy (HDCT) in patients who relapse and its outcome.

*N*	PF to HDCT	At first salvage	At second salvage	At third salvage
		*N* = 24	*N* = 29	*N* = 2
55	17 (31%)	12 (50%)	5 (17%)	0

Patients relapsing following high-dose chemotherapy were treated with a variety of chemotherapy regimens. The most common regimens included gemcitabine and paclitaxel and cisplatin and epirubicin—oral etoposide was used infrequently. Of the 38 patients who were not progression free to high-dose chemotherapy and received further therapy/therapies, there was 1 long-term survivor who remains disease free at 8 years having had VIP ×3, an RPLND which showed cancer and then gemcitabine and docetaxel as the last regimen.

### Changes in chemotherapy delivery across the network in response to results of supranetwork audits

Following audits, it was agreed that the cut-off to start BEP would be a neutrophil count of 0.5 × 10^9^/l and a platelet count of 75 × 10^9^/l. If these levels were not met, then treatment would be delayed by 48 h (rather than the week that had been the case in some centres). In addition, the routine use of fluoroquinolone prophylaxis during neutropenia was agreed.

It is clear that the SMDT discussion resulted in some reductions in the amount of chemotherapy given. Patients with stage 1 non-seminomatous germ cell tumour (NSGCT) were offered 1 cycle of BEP rather than 2 following the Albers study^16^ In addition, SMDT discussion ensured that 3 cycles of BEP rather than 4 cycles were given to everyone with IGCCCG good prognosis disease—previously 23 (2.7%) patients with slow marker decline (failure to decline to anticipated half-life after cycle 2 or beyond) had been given 4 cycles.

Retroperitoneal surgery was provided by two specialist sites. The number of patients undergoing this has remained stable. The number of patients referred for pulmonary, liver or brain surgery was relatively small—9 in 10 years, 6 of whom had teratoma or necrosis and 3 had active cancer.

### Domain 3: radiology

Three thousand two hundred and six cases were centrally reviewed. These included all stage 1 cases from LVCs. Stage 1 cases from HVCs were not automatically reviewed. All metastatic cases were centrally reviewed as were all relapsed cases. The main contribution of the centre radiologists was the expert interpretation of equivocal imaging, in addition to the small number of patients where staging was revised.

The most common change resulted when a residual mass showed an increase in size and the scan had been reported as progressive malignant disease. The context of the SMDT allowed an understanding that this may represent growing teratoma syndrome. A decrease in computed tomographic (CT) density and the appearance of calcification in the presence of falling tumour markers made pseudo-progression more likely and referral directly for retroperitoneal surgery was made (*n* = 20). In six cases, misinterpretation of blood vessels or other structures led to a report of progressive disease that was reversed by the SMDT. A total of 10 cases presented as metastatic disease were restaged as stage 1, with borderline nodes at atypical sites being the most frequent reason. In a further 14 cases, changes suggestive of alternative additional diagnoses were made (e.g. a thickened bladder wall, symmetrical hilar lymph node enlargement due to sarcoidosis). Thus significant changes to radiology were made in just under 3% of cases.

The role of ^18^F-fluorodeoxyglucose positron emission tomography with CT (^18^FDG PETCT) has evolved throughout this period. ^18^FDG PETCT was used for:

1. Rising markers without evidence of disease on CT.
2. Post chemotherapy for NSGCTs.
3. Post chemotherapy seminomatous masses >30 mm in diameter.
4. In patients who had retroperitoneal disease and pre-orchidectomy raised markers that had normalised following surgery or stage 1 disease, which had recurred on conventional CT without a rise in tumour marker.
5. Patients with teratoma on the orchidectomy specimen whose CT indicated recurrence with normal markers.

In the first 2 years, as ^18^FDG PET-CT was not routinely available, 9 ^18^FDG PET-CT scans were done prior to decision-making for 48 patients.

In the following 8 years, a total of 111 ^18^FDG PET-CT studies was planned, and 106 studies were performed for 185 patients who were being considered for an RPLND. In a further 65 patients, ^18^FDG PET-CT was not considered necessary by the SMDT and in 9 cases it was not known whether ^18^FDG PET-CT was planned.

Of the 106 ^18^FDG PET CT studies performed, 31 were clearly FDG avid, 59 were negative, 14 were mildly avid and 2 results were not known. In the 31 avid cases, 4 had surgery (2 had viable cancer and pathology was not known for the other 2), 23 had chemotherapy, 2 were observed and in 2 the outcome was unknown. In total, 24 patients received chemotherapy—23 with positive ^18^FDG PET-CT and 1 with mildly avid FDG uptake. Of these, 16 were treated on the findings of ^18^FDG PET-CT alone, 1 had biopsy-proven disease, 6 had raised tumour markers and 1 was already having chemotherapy and, based on the PET result, continued it.

In the 14 cases with mild avidity, 4 underwent an RPLND and all had teratoma/necrosis, 9 were observed and 1 had chemotherapy. Of those observed, 7 remained alive and well and 2 died of other causes.

In 59 cases where the ^18^FDG PET-CT scan was negative, surgery was planned for 29 but in 4 the markers increased before the surgery and they received further chemotherapy. Of the 25 who had surgery, 5 had viable cancer, 18 had teratoma/necrosis and in 2 the pathology was unknown. A further 28 were observed and 2 later relapsed and received further therapy. In two, the outcome was unknown.

**Fig. 1:**
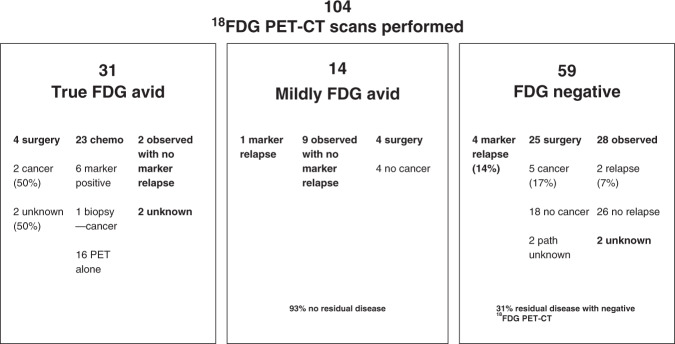
Summary of the ^18^FDG PET-CT results. Outcome of patients who had a ^18^FDG PET-CT scan as part of their post chemotherapy management for their germ cell tumours.

The data are summarised in Fig. 1 and shows, in the presence of a negative ^18^FDG PET-CT, 19% of those with a negative scan had residual malignant disease. In our data, these were surgically confirmed (*n* = 5) or displayed a marker recurrence pre-op (*n* = 4) or later relapse (*n* = 2). In a mildly positive ^18^FDG PET-CT, 7% had residual malignant disease with a marker relapse (*n* = 1). In true-positive ^18^FDG PET-CT, 13% (*n* = 4) had surgery and 2 had histological confirmed viable cancer and the other 2 were not known.

In the 65 patients where ^18^FDG PETCT was not thought to be helpful in making the decision for surgery, 61 had surgery and 47 of these had teratoma/necrosis and 14 (23%) had cancer. The remaining two were observed and two had chemotherapy.

For the 9 patients where it was unknown whether PET was planned, all had surgery, 6 had teratoma/necrosis and 3 had cancer.

Comparing the results of those who had a negative ^18^FDG PET-CT with those whom the SMDT felt the scan was not required and pathology and/or marker change was known, 14/61 (23%) who were not planned for ^18^FDG PET-CT had viable cancer in the RPLND specimen; 9/29 (31%) who had a negative scan and were planned for surgery had either viable cancer (*n* = 5) or marker rise immediately before the surgery was undertaken (*n* = 4). Based on the negative result, an additional 28 were observed and only 2 of these were known to relapse later.

In order to assess the predictive value of PET scans, the cases with unknown pathology post-surgery were excluded as were those cases where a positive PET scan led to the use of chemotherapy in the absence of a rise in markers. Using this approach, a total of 80 scans were evaluable. Using this approach, we accept that the sensitivity will be unrepresentatively low. Eleven were positive of whom 9 were true positive (positive predictive value 0.82). Sixty-nine were negative of whom 57 did not progress (negative predictive value of 0.83). Sensitivity of the test was 0.43 (9/21) and specificity was 0.97 (57/59).

### Domain 4: pathology

During the 10-year period, the number of cases undergoing central pathology review rose as an increasing awareness of the value of specialist uropathology review was appreciated. Initially 30–45 cases were centrally reviewed annually but by 2016 this had risen to 243. Overall, 790 cases were centrally reviewed with 75 changes (9.4%). Initially cases were reported by the local pathologist with selective referral for expert uropathologist review—this has changed with time and in the last 5 years—all specimens have been reviewed by an experienced uropathologist. These are predominantly based at the HVCs. In 20 cases, the pathological tumour, node and metastasis stage was modified: 14 cases were upstaged from pT1 to pT2/3 (e.g. due to the finding of lympho-vascular invasion) and 5 cases were down-staged from pT2/3 to pT1. One case with a discrete metastasis in the spermatic cord was upstaged to metastatic disease. In 5 cases, there was a complete change in the pathological diagnosis. Those cases are presented here:

1. Local report: benign; review—burnt out GCT.
2. Local report: spermatocytic tumour; review—high-grade malignancy—monocytic/plasmacytoid leukaemia.
3. Local report: parotid biopsy—germinoma; review—lymphoma.
4. Local report: orchidectomy—teratoma and cyst; review—no teratoma, but cystic dysplasia.
5. Local report: epididymal adenocarcinoma; review—adenocarcinoma of unknown primary.

In eight cases, i12P was used to confirm or refute a GCT primary. There were modifications to the tumour type in six cases: one case reported as pure seminoma was found to have an element of NSGCT, two reported as NSGCT were pure seminoma, and three reported as NSGCT were found to have an additional element of seminoma. In 12 other cases, the percentage of the various tumour elements were changed. There were nine patients who had complex pathology discussed, another six who required re-review and one patient who was found on review to have disease under the caecal mucosa. A further five patients had changes made to the RPLND specimen details, two patients had suboptimal immunohistochemistry done on the original pathology and one had a smaller tumour size than the original report.

### Domain 5: specialised cases

#### Patients with learning difficulties or mental health issues

Several areas came to light within this review. The number of patients with learning difficulties (those requiring an advocate for decision-making) or diagnosed with mental health problems such as schizophrenia was significant—26 patients fell into this group and 21/25 where staging was known (84%) presented with metastatic disease compared to 28% in our 10-year audit. Many of these patients had required a substantial amount of community support prior to diagnosis of a GCT. Managing their care provided significant challenges and longer stays in hospital were common. Informed consent of a vulnerable adult required best interest meetings and safeguarding support. Of these 26, 8 remain alive and well after treatment, 9 were offered treatment but the final decision to treat was not documented, 6 received treatment but follow-up information was insufficient and 3 have died after receiving >1 treatment. Another three were re-treated for relapsed disease—one is known to be alive and well and in the other two the outcome is unknown.

#### Very late relapse—beyond 10 years

There were 14 very late relapses at >10 years after initial diagnosis and treatment (range 13–36 years from last treatment). All relapsed from previously treated metastatic disease, had been discharged from routine follow-up and not all received their original treatments within our supranetwork. There are 7 still alive at >2 years since late relapse and only 1 of those has had a durable response without surgery emphasising the importance of surgery in the management of late relapses. Seven have died of disease. A raised α-fetoprotein (AFP) was detected at relapse in 8 patients and of these 6 have died and of the 2 who are alive 1 continues on treatment >2 years after his late relapse as he is not disease free. Of those who died, 5 relapsed with AFP >1000.^17^

#### Tumours in contralateral testis

Forty-one patients developed a tumour in their contralateral testis. The majority (71%) presented >5 years after original diagnosis and treatment (9 in <5 years and for 3 the interval was not known). Four patients were considered for either partial orchidectomy or to try pre-operative chemotherapy to try to save the testis but either the tumour was too large or the solitary testis non-functioning (low testosterone).

## Discussion

This comprehensive audit of the GCT SMDT has allowed the assessment of trends over a significant period of time.^18^ Use of a proforma has facilitated aspects of this audit supporting the findings of De Leso et al.^19^ The policy of minimal discussion for stage 1 cases and those achieving complete radiological remission after chemotherapy has allowed more time to be concentrated on the complex cases. Other audits of MDT working confirm the value of spending more time on complex cases.^19,20^

The data presented from an SMDT providing a service to a large population base suggests that it may be possible to achieve good results within small centres working as part of a large SMDT where cross referrals to large volume centres is made easy.^21^ The harmonising of thresholds of laboratory parameters of when it is safe to re-treat patients with chemotherapy, as well as other discussions regarding the use of supportive care, have allowed the results from the smaller centres to improve.^22^ This enables patients to be treated closer to home with the experience of care offered by larger SMDT numbers.

Video conferencing was used to connect the sites for a 1-h meeting every week. The quality of this was (and continues to be) very variable and sometimes made the meeting difficult. In addition, it could be difficult to be critical of anything done when a person was on video link. The annual meeting that lasted a whole day was very helpful in allowing everyone to meet face to face. It also ensured that trials could be publicised for the group—not all were opened at the smaller centres and it was clear that some patients remained unwilling to travel to a large centre for treatment.

The effect of SMDT working on the outcome at smaller centres is clearly encouraging. During the first 5 years of this audit, the outcome at smaller centres for good prognosis disease was significantly worse than the HVCs (87.8% vs 95.3% survival at 5 years). In the last 5 years, that difference has narrowed (93.3% vs 95.1%) and is no longer statistically significant—this supports the suggestion that closer working and tighter delivery of care can overcome the effect of limited centre size.^23,24^ The fact that the treatment is the same—BEP for a majority of patients—means there is less reason for variation. While Woldu et al.^22^ reported a volume of case and outcome relationship in testicular cancer, there is evidence from this series at least that the SMDT can be a focus to improve the outcome of smaller centres as an alternative to further centralisation of services.

Our outcomes can be compared with Indiana University^21^—where they demonstrated PFS at 5 years was 90, 84 and 54%, our 2-year PFS was 93, 72 and 51% (in the last 5 years of this audit) and an overall estimated 5-year survival of 97, 92 and 73% vs our 5-year survival of 95, 84 and 59%. In the Indiana series, nearly half the patients began therapy at Indiana University. They attributed their improvement compared to the SEER database to improved supportive care and aggressive post-chemotherapy surgery. What was striking was the rate of surgery, as 128/759 metastatic cases underwent post-chemotherapy surgery in our series (16.8%) compared to 36% in the Indiana series. Although more of their patients fell into the poor prognosis group, this is clearly not the whole explanation. A more aggressive surgical approach may be contributing to their better survival.

The question of the optimal treatment for poor prognosis disease remains unresolved. While two recent randomised trials have shown improved PFS with more intensive therapy initially, the evidence that outcome is improved overall is much softer.^15,25^ The network results suggest an improved outcome for these patients when treated with early dose intense chemotherapy but that effective salvage therapy has narrowed the gap compared to those patients who started with BEP (or VIP).^12^

Supraregional working has ensured that patients get access to high-dose chemotherapy—with 55 getting access to this treatment. Most were after two lines of conventional chemotherapy. Not surprisingly, the results seem better after only one line of chemotherapy.

It is clear that, even when treatment fails, further palliative treatments are being offered.^26^ Part of this reflects the young patient group. Disease-specific phase 1/2 studies are rare, and in the TYA group in particular, it is clearly very difficult for a parent to ‘give up’ as there is a feeling that all options of treatment should be explored first. Being aware of network early phase trials becomes valuable—some of these studies may permit entry of various tumour types.

This review of SMDT suggests that centralised pathology and radiology review can lead to important modifications in diagnostic reports. The review of ^18^FDG PET-CT scan use suggested that these scans may be much less useful than originally thought and that their ability to inform on the need for surgery has been overestimated. Recent data about the use of PET in residual masses post chemotherapy for seminoma suggests a similar message.^27^

The effect of pathology review is more difficult to quantify. Many of the modifications would not have affected outcome but may have led to changes in advice—e.g. whether to offer surveillance or adjuvant therapy and also the intensity of follow-up, which could make a significant difference to the individual patient. Modifications were made to 9.4% of cases, which is less than that reported in a smaller series by Purshouse et al.^28^

This audit shows that, outside the realm of clinical trials and HVC reporting, patients with particular challenges such as learning difficulties or other mental health problems and those with very late relapses each present problems that are less commonly addressed and do require tailored individualised treatment plans. Very late relapse are a particular problem as often these patients have a rising AFP and chemo-sensitivity is markedly reduced, making cure without surgery to remove all sites of disease very unlikely.^17^

## Conclusions

SMDT working has led to a refining of therapy across a large network. Changes in treatment administration mean that patients attending small centres no longer seem to be at a disadvantage in terms of OS. Pathology and radiology review have been helpful in small percentages of patients, but this is important where the OS is so good, and these changes can lead to a reduction in treatment. Further emphasis needs to be on the assessment of the burden of long-term side effects and the persisting poor outcome for patients with poor prognosis disease.^29^

## Data Availability

Data were collected from our standardised MDT discussion forms. The data sets used and/or analysed are available from the corresponding author on reasonable request.
